# A Systematic Review of Breast Cancer Knowledge Among School-Level Students Worldwide

**DOI:** 10.1007/s13187-025-02602-5

**Published:** 2025-03-19

**Authors:** Camila U. Malfatto, Gloria M. Calaf

**Affiliations:** 1 Pontificia Universidad Católica de Valparaíso, 2340025 Valparaiso, Chile; 2 Instituto de Alta Investigación, Universidad de Tarapacá, 1000000 Arica, Chile

**Keywords:** Breast cancer, Breast self-examination, Education, High school students, Educational interventions

## Abstract

Breast cancer knowledge and the practice of breast self-examination among school students are crucial topics in health education due to the high incidence rate. However, there is little research on this subject. This systematic review aims to synthesize the empirical literature on the topic, analyzing and identifying patterns in the learning strategies and evaluation methodologies used. A systematic search, following the PRISMA method across the Scopus, Web of Science, and ERIC databases, found only six studies published between 2000 and 2021 that focused on school education. The results revealed a significant lack of explicit and detailed educational interventions. Only two studies included educational interventions, but both lacked specific methodological details, limiting the ability to assess their effectiveness and replicate the results. While cross-sectional studies are useful for evaluating general knowledge, they did not sufficiently explore the underlying causes of knowledge deficiencies, nor did they propose strategies to address them. There is an urgent need to develop detailed and well-documented educational interventions on breast cancer within school contexts. This review provides an initial foundation and suggests that future research should address the identified methodological gaps to effectively contribute to health education and breast cancer prevention in young populations.

## Introduction

Breast cancer is a disease characterized by the uncontrolled proliferation of cells in the mammary gland, forming tumors that, if left untreated, can spread and become fatal [1]. According to the World Health Organization (WHO), 2.3 million new cases of breast cancer were diagnosed in 2022, and 670,000 people died from the disease, making it the leading cause of cancer death among women worldwide [2]. In Chile, the Department of Health Statistics and Information reported 1724 deaths from breast cancer in 2021, with a mortality rate of 8.76 per 100,000 inhabitants [3]. Despite the severity of breast cancer, specific education on this disease in the Chilean school curriculum is limited.

In the Chilean educational context, breast cancer is addressed indirectly in some biology and health science courses, but there are no specific educational objectives in the general curriculum [4]. This educational gap contrasts with the urgent need to improve breast cancer awareness and prevention, especially among adolescents. This review aims to answer the question: What do secondary school students know about breast cancer? How have these studies been conducted? Understanding breast cancer knowledge and self-care practices among secondary school students is crucial to developing effective teaching programs that will promote early detection and prevention.

The objective of this systematic review is to analyze and synthesize the existing literature on breast cancer knowledge and breast self-examination (BSE) in secondary school students worldwide, identifying patterns in learning strategies and evaluation methodologies. The specific objectives are as follows: (1) to analyze a representative sample of literature that includes empirical research on BSE and breast cancer knowledge in school-level students, (2) to identify patterns that help characterize the effective development of learning strategies related to breast cancer, and (3) to critically analyze the articles based on their coherence, design, and methodology. The findings of this review can contribute to improving health education and self-care practices among adolescents and help to reduce the incidence and mortality of breast cancer in the long term.

## Data Collection

This review followed the PRISMA guidelines (Preferred Reporting Items for Systematic Reviews and Meta-Analyses) [5], which allowed for transparent documentation of the review, the authors’ actions, and the findings obtained. The conducted systematic review was original and analyzed and synthesized the existing literature on breast cancer knowledge and BSE among secondary school students worldwide.

Specific inclusion and exclusion criteria were defined for the study selection. Included studies had to be empirical articles investigating breast cancer knowledge and breast self-examination in school-level students, published in indexed journals, and focused on adolescent participants (school students). Publications in both English and Spanish were considered. Excluded studies were those that focused solely on general breast cancer without specifically addressing knowledge among school students and those focused on Pap smears, nursing students, cervical cancer, and adults or young adults outside the school context. Non-indexed publications, theses, and unpublished reports were also excluded.

The consulted information sources included the Scopus, Web of Science (WoS), and ERIC databases, which were selected for their broad coverage and relevance in scientific and educational research. Specific search terms and combinations were used for each database:

- In Scopus, the search terms were as follows: “breast self-examination” OR “breast cancer self-examination” AND (adolescents) AND (school OR “school education” OR education).
- In Web of Science, the search criteria were as follows: TS = (“breast self-examination” OR “breast cancer self-examination”) AND TS = (adolescents) AND TS = (school OR “school education” OR education) AND DT = (Article).
- In ERIC, the search terms were as follows: “breast self-examination” OR “breast cancer self-examination” AND (adolescents) AND (school OR “school education” OR education).

**Fig. 1:**
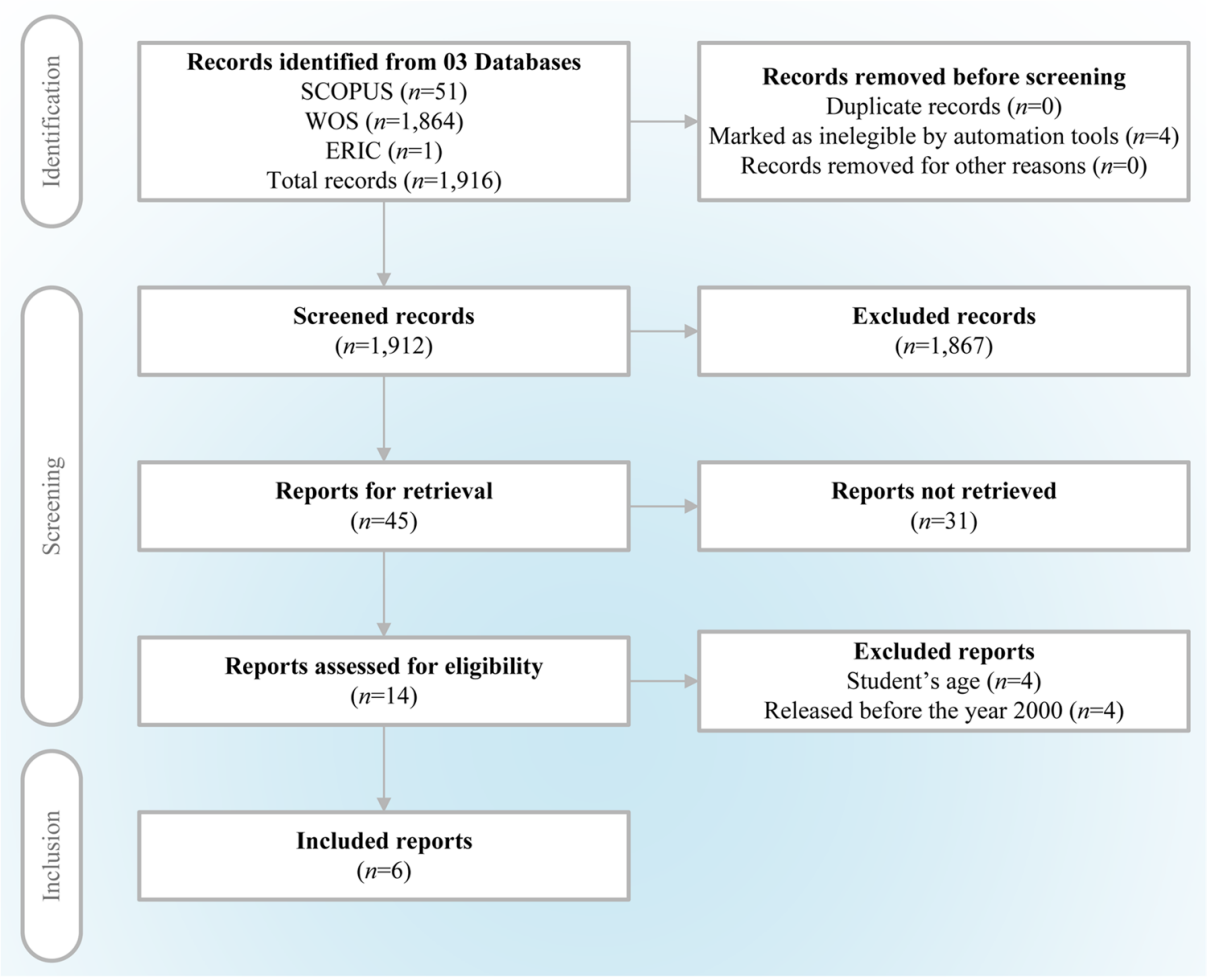
PRISMA flow diagram of the study selection process. The diagram shows the process of identification, screening, eligibility, and inclusion of studies for the systematic review. Adapted from the PRISMA model by Page et al. [6]

The article selection process followed several stages, as shown in the PRISMA [5] flow diagram (Fig. 1). First, 1916 potential article records were identified, of which 1912 were considered for initial screening. At this stage, 1867 articles were excluded for not meeting the inclusion criteria, leaving 45 articles for a more detailed review. However, 31 articles were excluded from full evaluation due to the age of the participants and the nature of the interventions observed in the abstract (some were not within the school context). Eventually, 14 articles were assessed for eligibility but only six studies were included in the final review since they were the only ones complying with the criteria for date selection (from the year 2000 onwards).

## Breast Cancer and Breast Self-Examination in Several Schools from Different Countries

Relevant data from the selected articles were extracted and summarized in Table 1. Then, a narrative synthesis was conducted to identify patterns and trends in the learning strategies and evaluation of breast cancer knowledge among secondary school students.

**Table 1:** Knowledge of breast cancer and breast self-examination in female students from secondary schools in several countries

Study design Participants (sex) Age at start Duration Reference	Objective	Results	Comments
Cross-sectional study 6380 female secondary school students from Jeddah Saudi Arabia Milaat et al. (2000) [7]	Females 6380 (secondary school students) To assess the knowledge and attitudes of secondary school students on breast cancer and breast self-examination (BSE)	Students had heard of BSE 2526/6380 (39.6%) Students have read scientific information about breast cancer 3004/6380 (47.1%) Breastfeeding is not a risk factor for breast cancer 3311/6380 (51.9%)	Data collection tool: Self-administered questionnaire Data analysis: SPSS, descriptive statistics, and chi-square tests Married students and those with children had higher levels of knowledge External researchers conducted the surveys
Cross-sectional study, quasi-experimental design 255 female ninth-grade students from four public secondary schools in Peoria, Illinois Ogletree et al. (2004) [8]	Females 112 control, 140 intervention group To evaluate the effectiveness of the BSE educational program presented to ninth-grade girls in public schools in Peoria Specific objectives: To determine whether knowledge about BSE and breast cancer increased because of the program and whether the program increased the intention to regularly perform breast self-examinations and the intention to encourage others to do the same	The school girls in the intervention group showed higher knowledge scores Number of students performing BSE before intervention: 11/112, 28/140 After: 10/112, 42/140	Data collection tool: 17-item questionnaire including multiple-choice, true/false, and yes/no questions. Intervention: 50-min BSE educational program including a PowerPoint presentation and a demonstration video Data analysis: SPSS v 11.5, Kruskal–Wallis test, and ANOVA The intervention was carried out by health education teachers from Midwest University
Cross-sectional study 200 female students from three all-girls secondary schools in Lagos, Nigeria Ages: between 12 and 18 years old Irurhe et al. (2012) [9]	Females 200 (120, 40, and 40 students randomly selected from three schools) To assess the level of awareness of breast cancer among secondary school students	Students had heard of breast cancer 194/200 (97%) Knowledge and understanding of the disease 108/200 (54%) Knowledge about breast cancer metastasis 102/200 (51%) Knowledge about BSE procedures ~ 100/200 (< 50%) BSE procedure should be done after menstruation 94/200 (47%) Knowledge about BSE technique using fingers 93/200 (46.5%) Inclusion of armpits in the BSE procedures 90/200 (45%)	Data collection tool: self-administered questionnaires conducted during recess. The questionnaire included three sections: demographics, awareness, knowledge of breast cancer, and BSE External researchers conducted the surveys
Cross-sectional study 800 adolescent female students from Colombo, Sri Lanka Ranasinghe et al. (2013) [10]	Females 800 (school students) To assess the knowledge, attitudes, and practices related to breast cancer, including detection methods, available services, breast self-examination (BSE), and sources of information among school adolescents in Sri Lanka	Deficiencies in knowledge, attitudes, and practices regarding breast cancer Knowledge of how to perform BSE 136/800 (17%) Recognition of mammography as an effective screening tool 248/800(35.6%) Chemotherapy as treatment for breast cancer 164/800(20.6%)	Students with special needs, learning disabilities, or disabilities were excluded from the sample External researchers conducted the surveys in 30 classes from 23 schools categorized as laboratory schools and four schools in the 1C category. Class selection was done through random sampling Data collection: Self-administered questionnaires available in Sinhala, Tamil, and English with 11 sections and 60 items (Data analysis: Univariate analysis using SPSS 18.0)
Cross-sectional study 432 female senior secondary school students from Anambra, Nigeria Ifediora and Azuike (2018) [11]	Females 321 (valid questionaries out of 432) To assess the knowledge, attitudes, and practices related to breast cancer and breast self-examination (BSE) among adolescent secondary school students in the Otuocha Educational Zone, to provide baseline information for early, targeted interventions	General knowledge of breast cancer 241/321 (75.2%) specific knowledge about risk factors 133/321 (41.5%) Specific knowledge on symptoms 147/321 (46.1%) Positive attitudes toward BSE 236/321 (73.6%) Performs BSE monthly 20/321 (6.1%) Never practiced BSE 177/321(55.3%)	Data collection tool: Self-administered questionnaire adapted from previous studies and validated with a pilot group of students. Data analysis: SPSS® version 25.0 using descriptive statistics and binary logistic regression External researchers surveyed 24 government schools (SSS-3) in the Otuocha Educational Zone
A quasi-experimental study with pre- and post-evaluation design 280 female students from Akure, Nigeria Ages: between 12 and 19 years old Six sessions (45 to 60 min) Ibitoye et al. (2021) [12]	Females 280 (Adolescent students) To assess the impact of education on the knowledge, attitude, and practice of breast self-examination (BSE) among adolescents	**Pre-evaluation**: Knowledge of breast cancer 193/280 (68.9%) How to perform BSE 71/280 (25.4%) Positive attitude toward BSE 94/280 (33.9%) Practice BSE before intervention 113/280 (40.4%) **Post-evaluation**: Knowledge of breast cancer 271/280 (97%) How to perform BSE 159/280 (56.8%) Positive attitude toward BSE 185/280 (66.1%) Practice BSE before intervention 224/280 (80%)	Participants were intentionally selected because they had developed breasts, fitting the purpose of the study Pre- and post-test evaluation. Six educational sessions on BSE lasting approximately 45 to 60 min, with 50 students per session. The program included PowerPoint presentations and videos on BSE, and practical demonstrations Data analysis: SPSS version 20, using descriptive statistics including frequencies, percentages, central tendency measures, chi-square tests, and independent *t*-tests, with a significance level (*p*-value) set at 0.05 External researchers conducted the survey and implemented the intervention

*BSE* breast self-examination

A cross-sectional study conducted in Jeddah, Saudi Arabia, involving 6380 female secondary school students, assessed the students’ knowledge of breast cancer and breast self-examination (BSE) (Table 1). The results showed that only 39.6% of the students had heard about BSE, and their understanding of the risk factors of breast cancer was very limited [7]. Over 80% of participants could not correctly answer half of the questions related to the disease. However, 47.1% reported having read or heard some scientific information about it, and only 51.9% understood that breastfeeding is not a risk factor for breast cancer. Married students and those with children demonstrated higher levels of knowledge, as did students with a family history of breast cancer or those who had undergone medical procedures related to the disease. The study used self-administered questionnaires to collect data, which were analyzed using SPSS with descriptive statistics and chi-square tests. These findings highlight the urgent need for educational programs aimed at promoting early detection and increasing awareness of breast cancer among secondary school students, a critical group for such interventions.

A quasi-experimental study (Table 1), involving 255 ninth-grade female students from four public high schools (140 in the intervention group and 112 in the control group) in Peoria, Illinois, was conducted [8]. The study assessed the effectiveness of a 50-min educational program on BSE. The program included a PowerPoint presentation covering breast cancer facts and BSE techniques and a demonstration video. The primary goal was to assess whether the program improved knowledge about BSE and breast cancer and whether it influenced the intention to perform BSE regularly and encourage others to do so. The results showed that students in the intervention group achieved higher knowledge scores and retained this knowledge over a five-to-six-week period than the control group. Additionally, the percentage of students performing BSE increased from 20 to 30% in the intervention group, while it slightly declined from 10 to 9% in the control group. There was also a significant improvement in the intention to perform BSE in the future among the students in the intervention group. The study used a 17-item questionnaire consisting of multiple-choice, true/false, and yes/no questions to gather data on knowledge and behavioral intent. Data were analyzed using SPSS v11.5, Kruskal–Wallis tests, and ANOVA. The findings support the feasibility of enhancing existing health education programs with content on breast self-examination.

The third study presented a cross-sectional analysis conducted in Lagos (Nigeria), involving 200 female students from three senior secondary schools, ages between 12 and 18 years old, who received self-administered questionaries about breast cancer general knowledge and BSE among other questions (Table 1). Each school had 600, 200, and 200 students, respectively; then, the sample selection process was done based on the ratios 3: 1: 1, that is, 120, 40, and 40 students were selected from each school, respectively. Such a selection process followed a systematic methodology based on a sample fraction of 5, i.e., the first member of the sample was selected randomly between the first and the fifth student of the school and from then on, the fifth student was selected to complete the sample [9]. Most students (97%) had heard about breast cancer; however, only 54% knew of the disease. Then, 37.5% of responders knew about the warning signs of breast cancer, 54.5% believed breast cancer patients could be cured if early detected, and 51% of students acknowledged that breast cancer could be metastasized. Then, 58.5% of students had heard about BSE as a technique for early detection of breast cancer; however, less than 50% were aware of the BSE procedure, 47% of responders knew such procedures were done after menstruation, 46.5% knew it was done using the tip of one’s fingers to feel the lumps, and 45% of students acknowledged that armpits should also be examined during the procedure.

The fourth study in Table 1 is a cross-sectional analysis conducted in Colombo, Sri Lanka, involving 800 adolescent female students [10]. Its objective was to assess knowledge, attitudes, and practices related to breast cancer, focusing on detection methods, available services, breast self-examination (BSE), and sources of information. Results revealed significant deficiencies in all the areas evaluated. Only 17.1% of the students knew how to perform BSE, and just 6.17% had attempted it. Additionally, 9.4% knew early detection methods, and only 5.5% knew where to seek help for breast cancer detection. The self-administered questionnaires, available in Sinhala, Tamil, and English, consisted of 11 sections and 60 questions covering demographics, risk factors, early warning signs, detection measures, and sources of information. The sample was selected through multi-stage stratified cluster sampling, and the data were analyzed using SPSS 18.0. Among the most notable findings, only 35.6% of the students recognized mammography as an effective screening tool, while 79.4% were unaware that chemotherapy is a treatment for breast cancer. Those with a family history of the disease demonstrated higher knowledge. This study highlights the urgent need to implement educational programs designed to address these gaps and promote positive health practices among adolescents in Sri Lanka, incorporating strategies that combine theoretical knowledge with practical approaches to foster greater awareness and long-term prevention.

The fifth study in Table 1 is a cross-sectional analysis conducted in the Otuocha Educational Zone, Anambra State, Nigeria, involving 432 senior secondary school students from 24 schools, of which 321 completed valid questionnaires [11]. The study aimed to evaluate the knowledge, attitudes, and practices related to breast cancer and breast self-examination (BSE) to provide baseline information for designing early and targeted interventions. The results revealed that only 4.1% reported a positive family history of breast cancer. While general knowledge about breast cancer was high (75.2%), specific knowledge about risk factors (41.5%) and symptoms (46.1%) was considerably low. 73.6% of the students expressed positive attitudes toward BSE; however, only 6.1% practiced it monthly, and 55.3% had never practiced it. The data collection tool was a self-administered questionnaire adapted from previous studies and validated with a pilot group of students. The study highlighted that the primary source of information about breast cancer was television or radio. The questionnaire covered demographic factors, knowledge about risk factors, symptoms, and BSE techniques, and related attitudes and practices. Data were analyzed using SPSS version 25.0, employing descriptive statistics and binary logistic regression. This study underscored the urgent need for health campaigns that not only promote BSE practices but also explain techniques, risk factors, and symptoms, emphasizing the correct methodology, timing, and frequency of BSE. The authors recommended incorporating BSE and breast cancer-related topics into school curricula, considering the positive attitudes observed among students suggest a strong potential for implementing effective and culturally appropriate educational programs.

The final study in Table 1 is a quasi-experimental design conducted at Fiwasaye Girls Grammar School in Akure, Nigeria, involving 280 adolescent students aged 12 to 19 years [12]. This study aimed to evaluate the impact of education on the knowledge, attitude, and practice of breast self-examination (BSE) among adolescents. Data were collected using a self-administered questionnaire administered both before and eight weeks after the intervention. The educational program consisted of six sessions, each lasting 45 to 60 min, with 50 students per session. It included PowerPoint presentations, demonstration videos, and guided practical activities on BSE. The results revealed significant improvements following educational intervention. Knowledge about breast cancer increased from 68.9% in the pre-test to 97.1% in the post-test. Similarly, the percentage of students who knew how to correctly perform BSE grew from 25.4 to 56.8%, and positive attitudes toward BSE increased from 33.9 to 66.1%. Regarding the practice of BSE, 40.4% of the students reported performing it before the intervention, while this percentage rose to over 80% afterward. Additionally, following the intervention, 79.3% of the students felt confident that they could accurately detect abnormalities in the breasts. Statistical analysis was performed using SPSS version 20, applying descriptive statistics, chi-square tests, and *t*-tests. This study highlighted the effectiveness of educational programs in improving knowledge, attitudes, and practices related to BSE among adolescents, emphasizing the importance of including such interventions in school health programs to promote early detection behaviors.

**Fig. 2:**
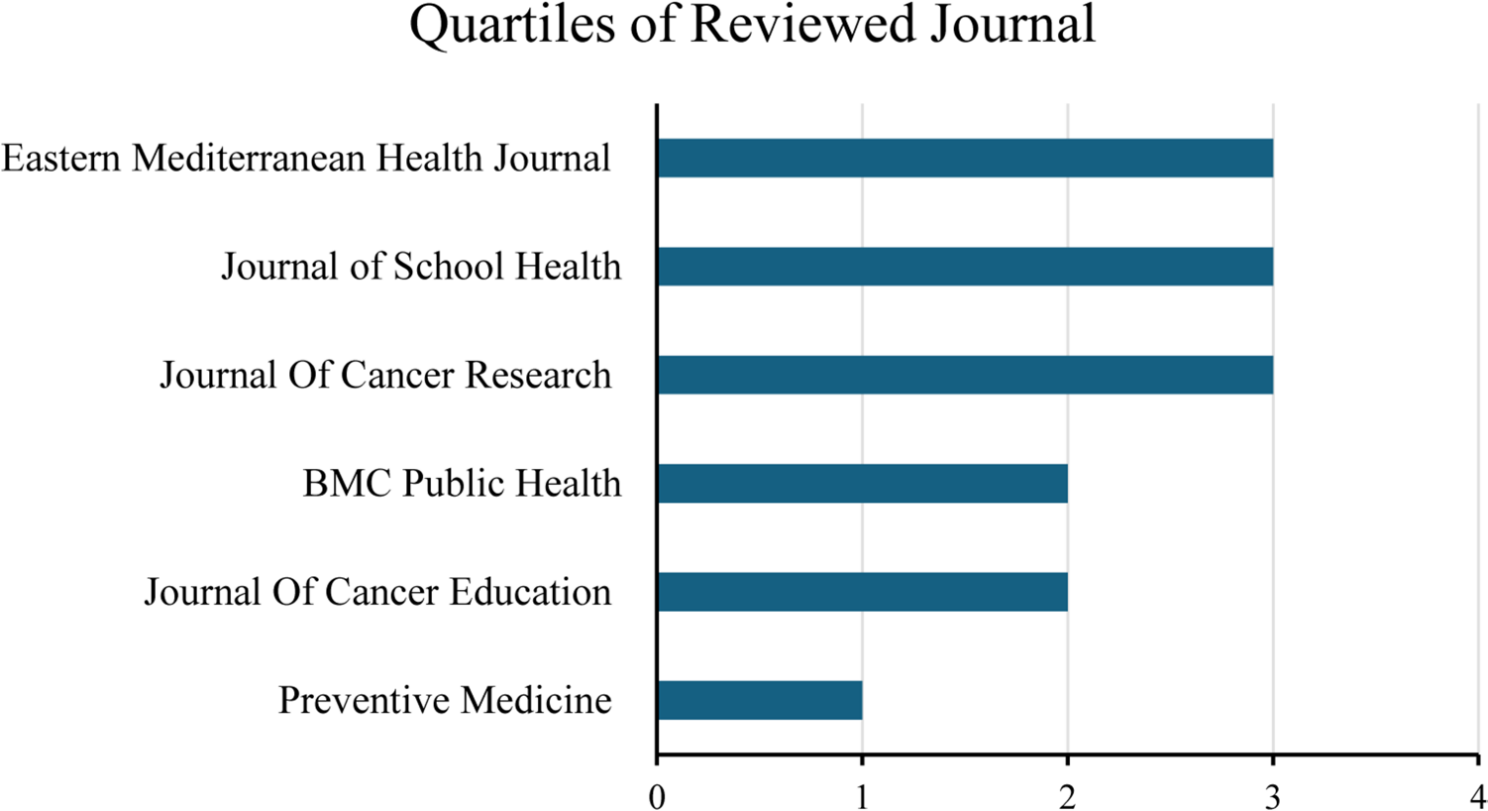
The graph shows the quartile distribution of the journals in this review. Each journal is categorized into one of four quartiles (Q1, Q2, Q3, and Q4), with Q1 representing the highest-ranked journals in terms of impact factor and Q4 representing the lowest. This classification reflects the influence of the journals and prestige in their respective fields, providing insight into the credibility and relevance of the studies published in them. Abbreviations, 1: Q1; 2: Q2; 3: Q3; 4: Q4

No articles written in Spanish related to the topic were identified in the consulted databases. After screening, six articles in English were selected to obtain a representative sample of empirical studies on breast cancer knowledge and BSE in secondary school students. The articles were published in six journals, with these journals having moderate to high impact levels, suggesting good recognition and credibility in their respective fields. This is reflected in their quartiles in Fig. 2 and impact factors in Table 2.

**Table 2:** Impact factor of the journals in the review

Journal	Impact factor
Eastern Mediterranean Health Journal	1.7
Journal of School Health	1.8
Journal of Cancer Research	2.7
BMC Public Health	3.5
Journal of Cancer Education	1.4
Preventive Medicine	4.3

## Discussion

Although research on breast cancer knowledge and practices among secondary school students is relatively scarce in terms of information, journal publications suggest that it is a topic of significant interest to the scientific and academic communities. Regarding the characteristics of the participants, they are divided into two groups: general secondary school students and students in specific contexts, such as all-girls schools or areas with low literacy rates. In terms of study design, two main groups were found: two studies with a quasi-experimental design, where data were collected before and after an intervention, and four studies with a cross-sectional design, where data were collected at a single point in time. When analyzing the number of participants, the studies ranged from 200 to 6380 participants, with cross-sectional studies having the largest sample sizes. Additionally, most participants were adolescent females. This suggests that the research is primarily focused on understanding breast cancer knowledge and BSE practices among young women, as they are a crucial group for breast health education interventions due to their high potential to adopt preventive practices. The prevalence of studies with large samples in cross-sectional designs indicates a significant effort to capture representative and relevant data that can influence educational policies and/or public health issues.

Regarding the relationship between the stated objectives and the materials and methods used, the data collection methods were mostly questionnaire-based, which aligns well with the objectives of evaluating knowledge and attitudes about breast cancer. All questionnaires included various items (breast cancer knowledge, self-examination knowledge, breast cancer warnings, etc.), allowing for a broad understanding of students’ knowledge. However, in four of the studies, self-administered questionnaires were used. While these allow for reaching many students, reducing costs, and providing privacy, they can respond anonymously. This approach had limitations, including the potential for ambiguous interpretation of questions, the lack of opportunity for clarification, the limited control over the conditions in which the questionnaires are completed, and the disregard for students’ context, which may lead to superficial responses. To achieve the objective, a focus group or including open-ended questions could be considered.

The two studies that included practical educational interventions reported by Ogletree et al. [9] and by Ibitoye et al. [12], indicated the materials used during the sessions, such as videos, PowerPoint presentations, or practical activities, but they did not elaborate on the specific content or teaching methodologies employed. This limits the ability to assess the effectiveness of these interventions or replicate them. Additionally, the lack of detailed information makes it difficult to understand the educational approach and its impact on the students. On the other hand, the statistical analysis appears solid and appropriate for measuring the impact of the interventions. Various programs and statistical tests were used, including SPSS (used in all studies for data analysis), non-parametric tests like Kruskal–Wallis, and ANOVA (to compare scores before and after the intervention), chi-square tests, *t*-tests (to examine associations and differences between variables), and binary logistic regression (to identify predictive factors).

Finally, regarding the results and conclusions, the analysis of cross-sectional studies seems coherent and highlights a lack of knowledge about breast cancer and self-care practices, given it is the leading cause of cancer-related death among women worldwide. However, none of the studies explored the underlying causes of this deficiency, nor did they address the educational, social, economic, or cultural barriers, nor did they propose strategies to overcome them. As for the studies that conducted educational interventions, the results were generally positive. Nevertheless, being an external and one-time intervention, there was no follow-up to determine whether this knowledge translated into sustained self-care behaviors. None of the interventions addressed how they could be adapted or implemented in different school contexts.

## Conclusion

This research conducted a critical and representative analysis of the literature that considered empirical research on breast self-examination and breast cancer knowledge among school-level students, aiming to identify patterns and characterize the effective development of learning strategies associated with breast cancer. The review revealed a lack of explicit and detailed educational interventions on breast cancer at the school level, and that adolescent populations had a low level of knowledge about both breast cancer and self-care practices. Few explicit educational interventions were found that could help identify effective learning strategies related to breast cancer, and among the countries with studies, only Nigeria, Saudi Arabia, the United States, and South Asia were represented. Only two of the studies involved educational interventions, and both lacked specific details about their methodologies. This limited the ability to assess and replicate these interventions, preventing the identification of consistent patterns. Therefore, future research must describe the educational content, strategies, and methodologies used. Comprehensive documentation will allow for a better understanding and development of effective learning strategies in breast cancer and self-care at the school level. The reviewed studies highlighted the lack of information and research on this topic worldwide, underscoring the importance of its inclusion in the curriculum of schools. While these studies provided an initial foundation on the subject, it is imperative that future research addresses the identified methodological deficiencies and focuses on generating new knowledge, thus contributing to early detection and developing self-care habits from a young age.

## Data Availability

Not applicable.

## Declarations

### Disclaimer

The funders had no role in the design of the study; in the collection, analyses, or interpretation of data; in the writing of the manuscript; or in the decision to publish the results.
